# Emergent vortices in populations of colloidal rollers

**DOI:** 10.1038/ncomms8470

**Published:** 2015-06-19

**Authors:** Antoine Bricard, Jean-Baptiste Caussin, Debasish Das, Charles Savoie, Vijayakumar Chikkadi, Kyohei Shitara, Oleksandr Chepizhko, Fernando Peruani, David Saintillan, Denis Bartolo

**Affiliations:** 1 Laboratoire de Physique de l'Ecole Normale Supérieure de Lyon, Université de Lyon and CNRS, 46, allée d'Italie, Lyon F-69007, France; 2 Department of Mechanical and Aerospace Engineering, University of California, San Diego, 9500 Gilman Drive, La Jolla, California 92093-0411, USA; 3 Department of Physics, Kyushu University 33, Fukuoka 812-8581, Japan; 4 Department for Theoretical Physics, Odessa National University, Dvoryanskaya 2, Odessa 65026, Ukraine; 5 Université Nice Sophia Antipolis, Laboratoire J.A. Dieudonné, UMR 7351 CNRS, Parc Valrose, Nice F-06108, France

## Abstract

Coherent vortical motion has been reported in a wide variety of populations including living organisms (bacteria, fishes, human crowds) and synthetic active matter (shaken grains, mixtures of biopolymers), yet a unified description of the formation and structure of this pattern remains lacking. Here we report the self-organization of motile colloids into a macroscopic steadily rotating vortex. Combining physical experiments and numerical simulations, we elucidate this collective behaviour. We demonstrate that the emergent-vortex structure lives on the verge of a phase separation, and single out the very constituents responsible for this state of polar active matter. Building on this observation, we establish a continuum theory and lay out a strong foundation for the description of vortical collective motion in a broad class of motile populations constrained by geometrical boundaries.

## Abstract (web summary)

Confined populations of interacting motile particles often display collective motion in the form of large-scale vortices, such as fish groups and bacteria colonies. Bricard *et al.* study a model system with self-propelled colloidal rollers and identify the constituents responsible for emergent vortices.

## Graphical abstract

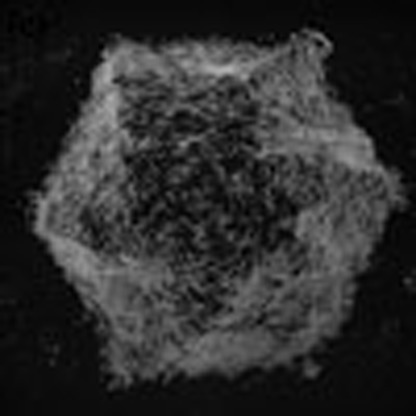

Building upon the pioneering work of Vicsek *et al.*^1^, physicists, mathematicians and biologists have contemplated the self-organization of living-organism groups into flocks as an emergent process stemming from simple interaction rules at the individual level^2^,^3^,^4^. This idea has been supported by quantitative trajectory analysis in animal groups^5^,^6^,^7^, together with a vast number of numerical and theoretical models^3^,^4^, and more recently by the observations of flocking behaviour in ensembles of non-living motile particles such as shaken grains, active colloids, and mixtures of biofilaments and molecular motors^8^,^9^,^10^,^11^,^12^. From a physicist's perspective, these various systems are considered as different instances of polar active matter, which encompasses any ensemble of motile bodies endowed with local velocity–alignment interactions. The current paradigm for flocking physics is the following. Active particles are persistent random walkers, which when dilute form a homogeneous isotropic gas. Upon increasing density, collective motion emerges in the form of spatially localized swarms that may cruise in a sea of randomly moving particles; further increasing density, a homogeneous polar liquid forms and spontaneously flows along a well-defined direction^1^,^13^,^14^. This picture is the outcome of experiments, simulations and theories mostly performed in unbounded or periodic domains.

Beyond this picture, significant attention has been devoted over the last five years to confined active matter^3^,^12^,^15^,^16^,^17^,^18^,^19^,^20^,^21^,^22^,^23^,^24^,^25^,^26^. Confined active particles have consistently, yet not systematically, been reported to self-organize into vortex-like structures. However, unlike for our understanding of flocking, we are still lacking a unified picture to account for the emergence and structure of such vortex patterns. This situation is mostly due to the extreme diversity in the nature and symmetries of the interactions between the active particles that have been hitherto considered. Do active vortices exist only in finite-size systems as in the case of bacterial suspensions^17^, which lose this beautiful order and display intermittent turbulent dynamics^27^ when unconfined? What are the necessary interactions required to observe and/or engineer bona fide stationary swirling states of active matter?

In this paper, we answer these questions by considering the impact of geometrical boundaries on the collective behaviour of motile particles endowed with velocity–alignment interactions. Combining quantitative experiments on motile colloids, numerical simulations and analytical theory, we elucidate the phase behaviour of *polar* active matter restrained by geometrical boundaries. We use colloidal rollers, which, unlike most of the available biological self-propelled bodies, interact via well-established dynamical interactions^11^. We first exploit this unique model system to show that above a critical concentration populations of motile colloids undergo a non-equilibrium phase transition from an isotropic gaseous state to a novel ordered state where the entire population self-organizes into a single heterogeneous steadily rotating vortex. This self-organization is *not* the consequence of the finite system size. Rather, this emergent vortex is a genuine state of polar active matter lying on the verge of a macroscopic phase separation. This novel state is the only ordered phase found when unidirectional directed motion is hindered by convex isotropic boundaries. We then demonstrate theoretically that a competition between alignment, repulsive interactions and confinement is necessary to yield large-scale vortical motion in ensembles of motile particles interacting via alignment interactions, thereby extending the relevance of our findings to a broad class of active materials.

## Results

### Experiments

**Figure 1:**
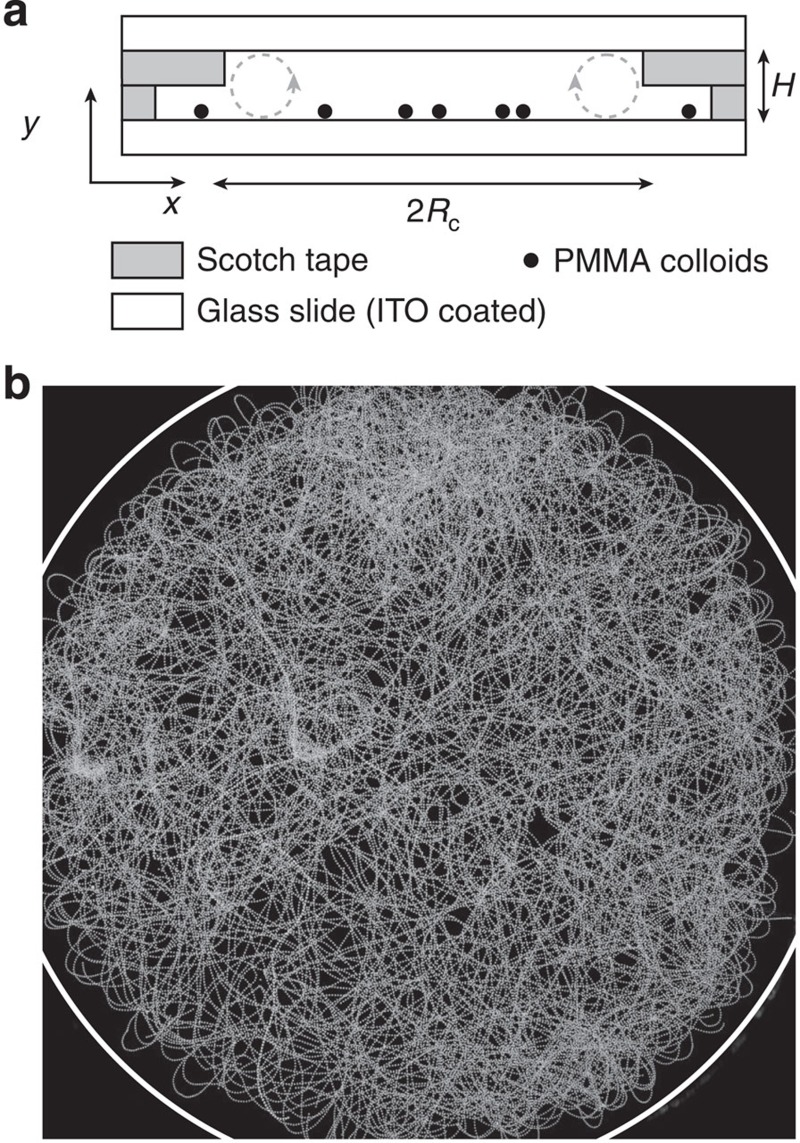
Experimental setup. (**a**) Sketch of the setup. Five5-micrometre PMMA colloids roll in a microchannel made of two ITO-coated glass slides assembled with double-sided scotch tape. An electrokinetic flow confines the rollers at the centre of the device in a circular chamber of radius *R*_c_. (**b**) Superimposed fluorescence pictures of a dilute ensemble of rollers (*E*_0_/*E*_*Q*_=1.1, *φ*_0_=6 × 10^−3^). The colloids propel only inside a circular disc of radius *R*_c_=1 mm and follow persistent random walks.

The experimental setup is fully described in the *Methods* section and in Fig. 1a,b. Briefly, we use colloidal rollers powered by the Quincke electrorotation mechanism as thoroughly explained in ref. 11. An electric field **E**_**0**_ is applied to insulating colloidal beads immersed in a conducting fluid. Above a critical field amplitude *E*_Q_, the symmetry of the electric charge distribution at the bead surface is spontaneously broken. As a result, a net electric torque acts on the beads causing them to rotate at a constant rate around a random axis transverse to the electric field^28^,^29^,^30^. When the colloids sediment, or are electrophoretically driven, onto one of the two electrodes, rotation is converted into a net rolling motion along a random direction. Here, we use poly(methyl methacrylate) (PMMA) spheres of radius *a*=2.4 μm immersed in a hexadecane solution.

As sketched in Fig. 1a, the colloids are handled and observed in a microfluidic device made of double-sided scotch tape and of two glass slides coated with an indium-tin-oxide layer. The ITO layers are used to apply a uniform DC field 
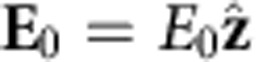
 in the *z*-direction, with *E*_0_=1.6 V μm^−1^ (*E*_0_=1.1*E*_Q_). Importantly, the electric current is nonzero solely in a disc-shaped chamber at the centre of the main channel. As exemplified by the trajectories shown in Fig. 1b and in Supplementary Movie 1, Quincke rotation is hence restrained to this circular region in which the rollers are trapped. We henceforth characterize the collective dynamics of the roller population for increasing values of the colloid packing fraction *φ*_0_.

### Individual self-propulsion

**Figure 2:**
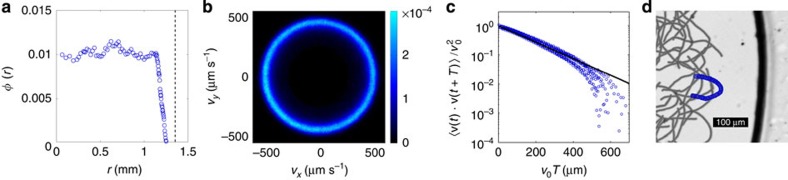
Dynamics of an isolated colloidal roller. (**a**) Local packing fraction *φ*(*r*), averaged over the azimuthal angle *φ*, plotted as a function of the radial distance. The dashed line indicates the radius of the circular chamber. (**b**) Probability distribution function of the roller velocities measured from the individual tracking of the trajectories. (**c**) Autocorrelation of the roller velocity 〈**v**_*i*_(*t*)·**v**_*i*_(*t*+*T*)〉 plotted as a function of *v*_0_*T* for packing fractions ranging from *φ*_0_=6 × 10^−3^ to *φ*_0_=10^−2^. Full line: best exponential fit. (**d**) Superimposed trajectories of colloidal rollers bouncing off the edge of the confining circle. Time interval: 5.3 ms (*E*_0_/*E*_*Q*_=1.1, *φ*_0_=6 × 10^−3^). Same parameters for the four panels: *R*_c_=1.4 mm, *E*_0_/*E*_*Q*_=1.1, *φ*_0_=6 × 10^−3^.

For area fractions smaller than 
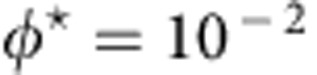
, the ensemble of rollers uniformly explores the circular confinement as illustrated by the flat profile of the local packing fraction averaged along the azimuthal direction *φ*(*r*) in Fig. 2a. The rollers undergo uncorrelated persistent random walks as demonstrated in Fig. 2b,c. The probability distribution of the roller velocities is isotropic and sharply peaked on the typical speed *v*_0_=493±17 μm s^−1^. In addition, the velocity autocorrelation function decays exponentially at short time as expected from a simple model of self-propelled particles having a constant speed *v*_0_ and undergoing rotational diffusion with a rotational diffusivity *D*^−1^=0.31±0.02 s that hardly depends on the area fraction (see Supplementary Note 1). These quantities correspond to a persistence length of 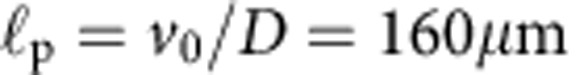 that is about a decade smaller than the confinement radius *R*_c_ used in our experiments: 0.9 mm<*R*_c_<1.8 mm.

At long time, because of the collisions on the disc boundary, the velocity autocorrelation function sharply drops to 0 as seen in Fig. 2c. Unlike swimming cells^26^,^31^, self-propelled grains^8^,^22^,^23^ or autophoretic colloids^32^, dilute ensembles of rollers do not accumulate at the boundary. Instead, they bounce off the walls of this virtual box as shown in a close-up of a typical roller trajectory in Fig. 2d, and in the Supplementary Movie 1. As a result, the outer region of the circular chamber is depleted, and the local packing fraction vanishes as *r* goes to *R*_c_, Fig. 2a. The repulsion from the edges of the circular hole in the microchannel stems from another electrohydrodynamic phenomenon^33^. When an electric field is applied, a toroidal flow sketched in Fig. 1a is osmotically induced by the transport of the electric charges at the surface of the insulating adhesive films. Consequently, a net inward flow sets in at the vicinity of the bottom electrode. As the colloidal rollers are prone to reorient in the direction of the local fluid velocity^11^, this vortical flow repels the rollers at a distance typically set by the channel height *H* while leaving unchanged the colloid trajectories in the centre of the disc. This electrokinetic flow will be thoroughly characterized elsewhere.

### Collective motion in confinement

**Figure 3:**
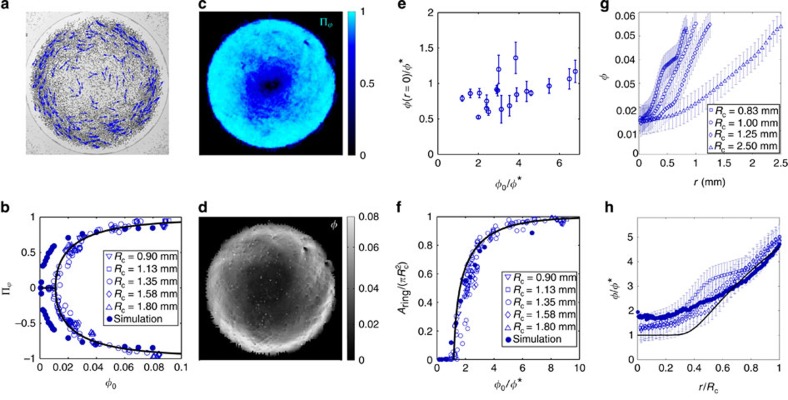
Collective-dynamics experiments. (**a**) Snapshot of a vortex of rollers. The dark dots show the position of one half of the ensemble of rollers. The blue vectors represent their instantaneous speed (*R*_c_=1.35 mm, *φ*_0_=5 × 10^−2^). (**b**) Average polarization plotted versus the average packing fraction for different confinement radii. Open symbols: experiments. Full line: best fit from the theory. Filled circles: numerical simulations (*b*=3*a*, *R*_c_=1 mm). (**c**) Time-averaged polarization field (*R*_c_=1.35 mm, *φ*_0_=5 × 10^−2^). (**d**) Time average of the local packing fraction (*R*_c_=1.35 mm, *φ*_0_=5 × 10^−2^). (**e**) Time-averaged packing fraction at the centre of the disc, normalized by 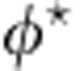 and plotted versus the average packing fraction. Error bars: one standard deviation. (**f**) Fraction of the disc where 
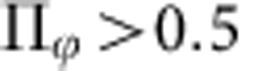
 versus the average packing fraction. Open symbols: experiments. Full line: theoretical prediction with no free fitting parameter. Filled circles: numerical simulations (*b*=3*a*, *R*_c_=1 mm). (**g**) Radial density profiles plotted as a function of the distance to the disc centre *r*. All the experiments correspond to *φ*_0_=0.032±0.002, error bars: 1*σ*. (**h**) Open symbols: same data as in **g**. The radial density profiles are rescaled by 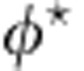 and plotted versus the rescaled distance to the centre *r*/*R*_c_. All the profiles are seen to collapse on a single master curve. Filled symbols: Numerical simulations. Solid line: theoretical prediction. All the data correspond to *E*_0_/*E*_*Q*_=1.1.

As the area fraction is increased above 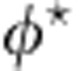, collective motion emerges spontaneously at the entire population level. When the electric field is applied, large groups of rollers akin to the band-shaped swarms reported in^11^ form and collide. However, unlike what was observed in periodic geometries, the colloidal swarms are merely transient and ultimately self-organize into a single vortex pattern spanning the entire confining disc as shown in Fig. 3a and Supplementary Movie 2. Once formed, the vortex is very robust, rotates steadily and retains an axisymmetric shape. To go beyond this qualitative picture, we measured the local colloid velocity field **v**(**r**, *t*) and use it to define the polarization field **Π**(**r**, *t*)≡**v**/*v*_0_, which quantifies local orientational ordering. The spatial average of **Π** vanishes when a coherent vortex forms, therefore we use its projection 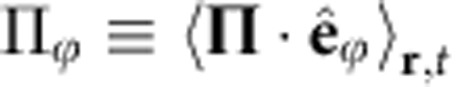 along the azimuthal direction as a macroscopic order parameter to probe the transition from an isotropic gas to a polar-vortex state. As illustrated in Fig. 3b, 
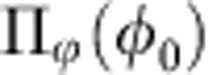
 displays a sharp bifurcation from an isotropic state with 
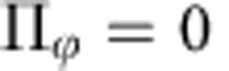
 to a globally ordered state with equal probability for left- and right-handed vortices above 
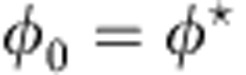
. Furthermore, Fig. 3b demonstrates that this bifurcation curve does not depend on the confinement radius *R*_c_. The vortex pattern is spatially heterogeneous. The order parameter and density fields averaged over time are displayed in Fig. 3c,d, respectively. At first glance, the system looks phase-separated: a dense and ordered polar-liquid ring where all the colloids cruise along the azimuthal direction encloses a dilute and weakly ordered core at the centre of the disc. We shall also stress that regardless of the average packing fraction, the packing fraction in the vortex core is measured to be very close to 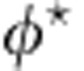, the average concentration below which the population is in a gaseous state, see Fig. 3e. This phase-separation picture is consistent with the variations of the area occupied by the ordered outer ring, *A*_ring_, for different confinement radii *R*_c_, as shown in Fig. 3e. We define *A*_ring_ as the area of the region where the order parameter exceeds 0.5, and none of the results reported below depend on this arbitrary choice for the definition of the outer-ring region. *A*_ring_ also bifurcates as *φ*_0_ exceeds 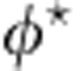, and increases with *R*_c_. Remarkably, all the bifurcation curves collapse on a single master curve when *A*_ring_ is rescaled by the overall confinement area 
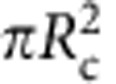
, Fig. 3f. In other words, the strongly polarized outer ring always occupies the same area fraction irrespective of the system size, as would a molecular liquid coexisting with a vapour phase at equilibrium. However, if the system were genuinely phase-separated, one should be able to define an interface between the dense outer ring and the dilute inner core, and this interface should have a constant width. This requirement is not borne out by our measurements. The shape of the radial density profiles of the rollers in Fig. 3g indeed makes it difficult to unambiguously define two homogeneous phases separated by a clear interface. Repeating the same experiments in discs of increasing radii, we found that the density profiles are self-similar, Fig. 3h. The width of the region separating the strongly polarized outer ring from the inner core scales with the system size, which is the only characteristic scale of the vortex patterns. The colloidal vortices therefore correspond to a monophasic yet spatially heterogeneous liquid state.

To elucidate the physical mechanisms responsible for this intriguing structure, we now introduce a theoretical model that we solve both numerically and analytically.

### Numerical simulations

The Quincke rollers are electrically powered and move in a viscous fluid, and hence interact at a distance both hydrodynamically and electrostatically. In ref. 11, starting from the Stokes and Maxwell equations, we established the equations of motion of a dilute ensemble of Quincke rollers within a pairwise additive approximation. When isolated, the *i*th roller located at **r**_*i*_ moves at a speed *v*_0_ along the direction 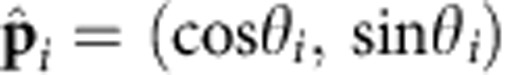 opposite to the in-plane component of the electrostatic dipole responsible for Quincke rotation^11^. When interacting via contact and electrostatic repulsive forces, the roller velocity and orientation are related by:


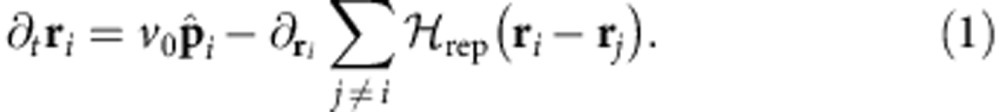


Inertia is obviously ignored, and for the sake of simplicity we model all the central forces acting on the colloids as an effective hard-disc exclusion 
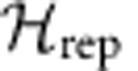
 of range *b*. In addition, *θ*_*i*_ follows an overdamped dynamics in an effective angular potential capturing both the electrostatic and hydrodynamic torques acting on the colloids^11^:


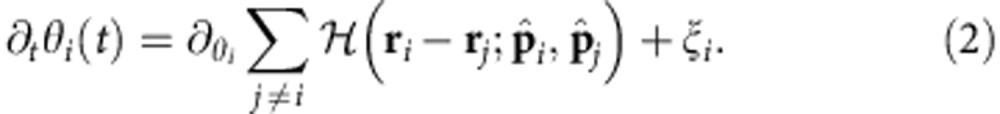


The *ξ*_*i*_'s account for rotational diffusion of the rollers. They are uncorrelated white noise variables with zero mean and variance 〈*ξ*_*i*_(*t*)*ξ*_*j*_(*t*′)〉=2*Dδ*(*t*−*t*′)*δ*_*ij*_. The effective potential in equation 2 is composed of three terms with clear physical interpretations:


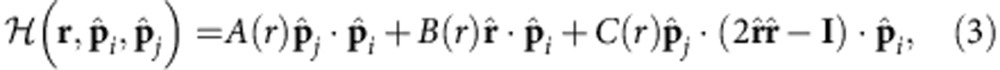


where 
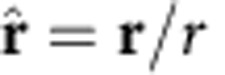
 and **I** is the identity matrix. The symmetry of these interactions is not specific to colloidal rollers and could have been anticipated phenomenologically exploiting both the translational invariance and the polar symmetry of the surface-charge distribution of the colloids^34^. The first term promotes alignment and is such that the effective potential is minimized when interacting rollers propel along the same direction. *A*(*r*) is positive, decays exponentially with *r*/*H*, and results both from hydrodynamic and electrostatic interactions. The second term gives rise to repulsive *torques*, and is minimized when the roller orientation points away from its interacting neighbour. *B*(*r*) also decays exponentially with *r*/*H* but solely stems from electrostatics. The third term has a less intuitive meaning, and promotes the alignment of 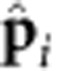 along a dipolar field oriented along 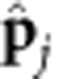. This term is a combination of hydrodynamic and electrostatic interactions, and includes a long-ranged contribution.

The functions *A*(*r*), *B*(*r*) and *C*(*r*) are provided in the Supplementary Note 2. As it turns out, all the physical parameters (roller velocity, field amplitude, fluid viscosity, etc.) that are needed to compute their exact expressions have been measured, or estimated up to logarithmic corrections, see Supplementary Note 2. We are then left with a model having a single free parameter that is the range, *b*, of the repulsive *forces* between colloids. We numerically solved this model in circular simulation boxes of radius *R*_c_ with reflecting boundary conditions using an explicit Euler scheme with adaptive time-stepping. All the numerical results are discussed using the same units as in the experiments to facilitate quantitative comparisons.

**Figure 4:**
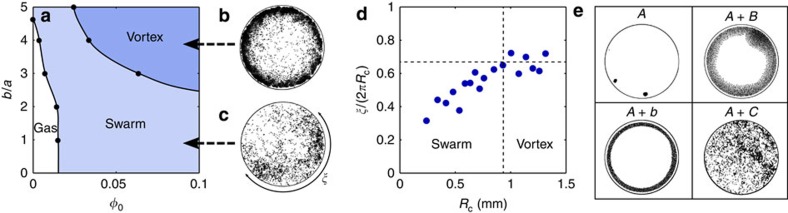
Collective-dynamics simulations. (**a**) The numerical phase diagram of the confined population is composed of three regions: isotropic gas (low *φ*_0_, small *b*), swarm coexisting with a gaseous phase (intermediate *φ*_0_ and *b*) and vortex state (high *φ*_0_ and *b*). *R*_c_=0.5 mm. (**b**) Snapshot of a vortex state. Numerical simulation for *φ*_0_=0.1 and *b*=5*a*. (**c**) Snapshot of a swarm. Numerical simulation for *φ*_0_=4.5 × 10^−2^ and *b*=2*a*. (**d**) Variation of the density correlation length as a function of *R*_c_. Above *R*_c_=1 mm, ξ plateaus and a vortex is reached (*φ*_0_=3 × 10^−2^, *b*=3*a*). (**e**) Four numerical snapshots of rollers interacting via: alignment interactions only (*A*), alignment interactions and repulsive torques (*A*+*B*, where the magnitude of *B* is five times the experimental value), alignment and excluded volume interactions (*A*+*b*, where the repulsion distance is *b*=5*a*), alignment and the *C*-term in equation 3 (*A*+*C*). Polarized vortices emerge solely when repulsive couplings exist (*A*+*B* and *A*+*b*).

The simulations revealed a richer phenomenology than the experiments, as captured by the phase diagram in Fig. 4a corresponding to *R*_c_=0.5 mm. By systematically varying the range of the repulsive forces and the particle concentration, we found that the (*φ*_0_, *b*) plane is typically divided into three regions. At small packing fractions, the particles hardly interact and form an isotropic gaseous phase. At high fractions, after a transient dynamics strikingly similar to that observed in the experiments, the rollers self-organize into a macroscopic vortex pattern, Fig. 4b and Supplementary Movie 3. However, at intermediate densities, we found that collective motion emerges in the form of a macroscopic swarm cruising around the circular box through an ensemble of randomly moving particles, Fig. 4c and Supplementary Movie 4. These swarms are akin to the band patterns consistently reported for polar active particles at the onset of collective motion in periodic domains^11^,^14^. This seeming conflict between our experimental and numerical findings is solved by looking at the variations of the swarm length *ξ*_s_ with the confinement radius *R*_c_ in Fig. 4d. We define *ξ*_s_ as the correlation length of the density fluctuations in the azimuthal direction. The angular extension of the swarms *ξ*_s_/*R*_c_ increases linearly with the box radius. Therefore, for a given value of the interaction parameters, there exists a critical box size above which the population undergoes a direct transition from a gaseous to an axisymmetric vortex state. For *b*=3*a*, which was measured to be the typical interparticle distance in the polar liquid state^11^, this critical confinement is *R*_c_=1 mm. This value is close to the smallest radius accessible in our experiments where localized swarms were never observed, thereby solving the apparent discrepancy with the experimental phenomenology.

More quantitatively, we systematically compare our numerical and experimental measurements in Fig. 3b,c for *R*_c_=1 mm. Even though a number of simplifications were needed to establish equations 1, 2 and 3 (ref. 11), the simulations account very well for the sharp bifurcation yielding the vortex patterns as well as their self-similar structure. This last point is proven quantitatively in Fig. 3h, which demonstrates that the concentration increases away from the vortex core, where 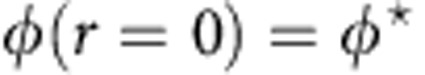, over a scale that is solely set by the confinement radius. We shall note however that the numerical simulations underestimate the critical packing fraction 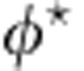 at which collective motion occurs, which is not really surprising given the number of approximations required to establish the interaction parameters in the equations of motion equation 3. We unambiguously conclude from this set of results that equations 1, 2 and 3 include all the physical ingredients that chiefly dictate the collective dynamics of the colloidal rollers. We now exploit the opportunity offered by the numerics to turn on and off the four roller-roller interactions one at a time, namely the alignment torque, *A*, the repulsion torque *B* and force *b*, and the dipolar coupling *C*. Snapshots of the resulting particle distributions are reported in Fig. 4e. None of these four interactions alone yields a coherent macroscopic vortex. We stress that when the particles solely interact via pairwise-additive alignment torques, *B*=*C*=*b*=0, the population condenses into a single compact polarized swarm. Potential velocity-alignment interactions are *not* sufficient to yield macroscopic vortical motion. We evidence in Fig. 4e (top-right and bottom-left panels) that the combination of alignment (*A*≠0) and of repulsive interactions (*B*≠0 and/or *b*≠0) is necessary and sufficient to observe spontaneously flowing vortices.

### Analytical theory

Having identified the very ingredients necessary to account for our observations, we can now gain more detailed physical insight into the spatial structure of the vortices by constructing a minimal hydrodynamic theory. We start from equations 1, 2 and 3, ignoring the *C* term in equation 3. The model can be further simplified by inspecting the experimental variations of the individual roller velocity with the local packing fraction, see Supplementary Fig. 1. The roller speed only displays variations of 10% as *φ*(**r**) increases from 10^−2^ to 4 × 10^−2^. These minute variations suggest ignoring the contributions of the repulsive forces in equation 1, and solely considering the interplay between the alignment and repulsion torques on the orientational dynamics of equation 2. These simplified equations of motion are coarse-grained following a conventional kinetic-theory framework reviewed in^4^ to establish the equivalent to the Navier-Stokes equations for this two-dimensional active fluid. In a nutshell, the two observables we need to describe are the local area fraction φ and the local momentum field *φ***Π**. They are related to the first two angular moments of the one-particle distribution function 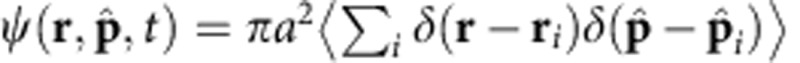, which evolves according to a Fokker-Plank equation derived from the conservation of 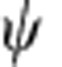 and equations 1 and 2. This equation is then recast into an infinite hierarchy of equations for the angular moments of 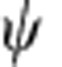. The two first equations of this hierarchy, corresponding to the mass conservation equation and to the momentum dynamics, are akin to the continuous theory first introduced phenomenologically by Toner and Tu^2^,^4^:


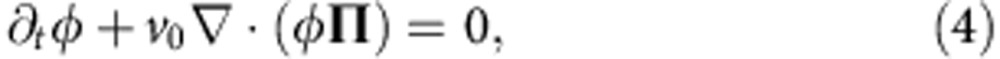



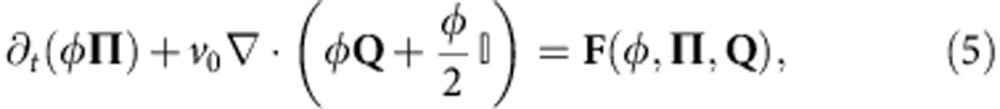


where **Q** is the usual nematic order parameter. The meaning of the first equation is straightforward, while the second calls for some clarifications. The divergence term on the left-hand side of equation 5 is a convective kinematic term associated with the self-propulsion of the particles. The force field **F** on the right-hand side would vanish for non-interacting particles. Here, at first order in a gradient expansion, **F** is given by:


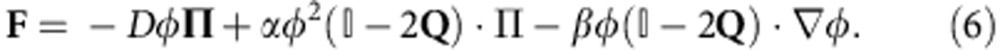


This force field has a clear physical interpretation. The first term reflects the damping of the polarization by the rotational diffusion of the rollers. The second term, defined by the time rate *α*=(∫_*r*>2*a*_*rA*(*r*)d*r*)/*a*^2^, echoes the alignment rule at the microscopic level and promotes a nonzero local polarization. The third term, involving *β*=(∫_*r*>2*a*_*r*^2^*B*(*r*)d*r*)/(2*a*^2^), is an anisotropic pressure reflecting the repulsive interactions between rollers at the microscopic level. equations 4 and 5 are usually complemented by a dynamical equation for **Q** and a closure relation. This additional approximation, however, is not needed to demonstrate the existence of vortex patterns and to rationalize their spatial structure.

Looking for axisymmetric steady states, it readily follows from mass conservation, equation 4, that the local fields must take the simple forms: *φ*=*φ*(*r*), 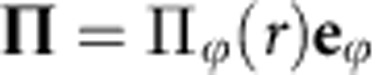 and 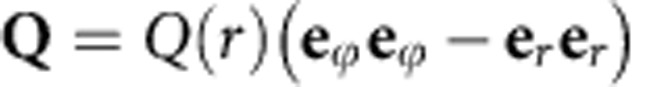 where *Q*(*r*)>0. We also infer the relation 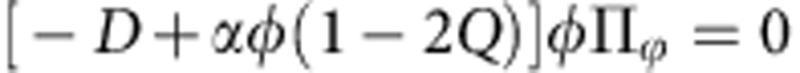 from the projection of the momentum equation, equation 5, on the azimuthal direction. This relation tells us that the competition between rotational diffusion and local alignment results in a mean-field transition from an isotropic state with 
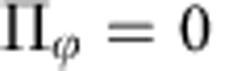
 to a polarized vortex state with 
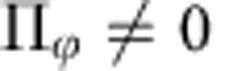
 and *Q*=(1)/(2)(1−*D*/(*αφ*)). This transition occurs when φ exceeds 
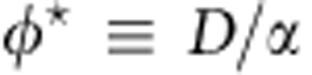
, the ratio of the rotational diffusivity to the alignment strength at the hydrodynamic level. In addition, the projection of equation 5 on the radial direction sets the spatial structure of the ordered phase:


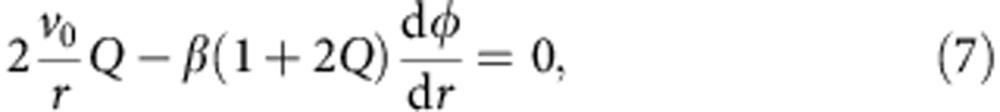


with again 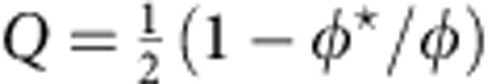 in the ordered polar phase. This equation has a clear physical meaning and expresses the balance between the centrifugal force arising from the advection of momentum along a circular trajectory and the anisotropic pressure induced by the repulsive interactions between rollers. It has an implicit solution given by


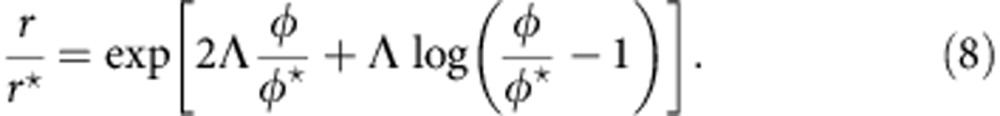


*φ*(*r*) is therefore parametrized by the dimensionless number 
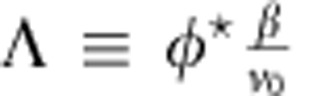
 reflecting the interplay between self-propulsion and repulsive interactions. Given the experimental values of the microscopic parameters, Λ is much smaller that unity 
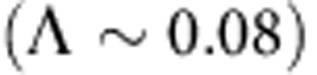
. An asymptotic analysis reveals that 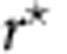 is the typical core radius of the vortex. For 
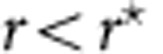
, the density increases slowly as 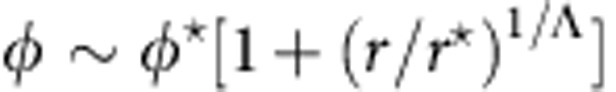 for all *φ*_0_ and *R*_c_. As *r* reaches 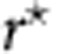, it increases significantly and then grows logarithmically as 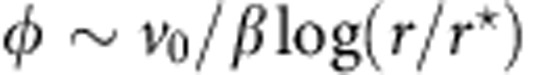 away from the vortex core. However, 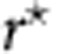 is an integration constant, which is solely defined via the mass conservation relation: 
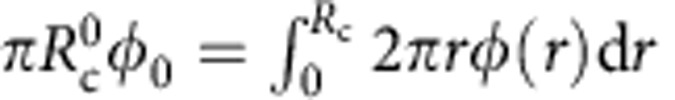
 and therefore only depends on *φ*_0_ and *R*_c_. 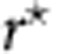 does not provide any intrinsic structural scale, and the vortex patterns formed in different confinements are predicted to be self-similar in agreement with our experiments and simulations despite the simplification made in the model, Fig. 3e. In addition, equation 8 implies that the rollers self-organize by reducing their density at the centre of the vortex down to 
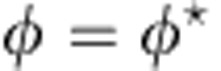
, the mean area fraction at the onset of collective motion, again in excellent agreement with our measurements in Fig. 3e.

To characterize the orientational structure of the vortices, an additional closure relation is now required. The simplest possible choice consists in neglecting correlations of the orientational fluctuations, and therefore assuming 
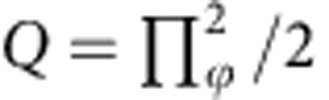
. This choice implies that


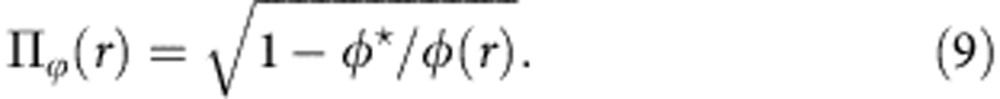


Equations 8 and 9 provide a very nice fit of the experimental polarization curve as shown in Fig. 3b, and therefore capture both the pitchfork bifurcation scenario at the onset of collective motion and the saturation of the polarization at high packing fractions. The best fit is obtained for values of 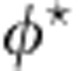 and *β*, respectively, five and two times larger than those deduced from the microscopic parameters. Given the number of simplifications needed to establish both the microscopic and hydrodynamic models, the agreement is very convincing. We are then left with a hydrodynamic theory with no free fitting parameter, which we use to compute the area fraction of the outer polarized ring where 
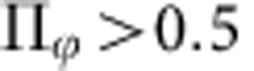
. The comparison with the experimental data in Fig. 3f is excellent.

Furthermore, equations 8 and 9 predict that the rollers are on the verge of a phase separation. If the roller fraction in the vortex core were smaller 
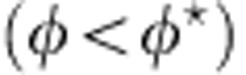
, orientational order could not be supported and an isotropic bubble would nucleate in a polar liquid. This phase separation is avoided by the self-regulation of *φ*(*r*=0) at 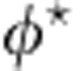.

## Discussion

Altogether our theoretical results confirm that the vortex patterns stem from the interplay between self-propulsion, alignment, repulsion and confinement. Self-propulsion and alignment interactions promote a global azimuthal flow. The repulsive interactions prevent condensation of the population on the geometrical boundary and allow for extended vortex patterns. If the rollers were not confined, the population would evaporate as self-propulsion induces a centrifugal force despite the absence of inertia.

We close this discussion by stressing on the generality of this scenario. First, the vortex patterns do not rely on the perfect rotational symmetry of the boundaries. As illustrated in Supplementary Fig. 2, the same spatial organization is observed for a variety of convex polygonal geometries. However, strongly anisotropic, and/or strongly non-convex confinements can yield other self-organized states such as vortex arrays, which we will characterize elsewhere. Second, neither the nature of the repulsive couplings nor the symmetry of the interactions yielding collective motion are crucial, thereby making the above results relevant to a much broader class of experimental systems. For instance, self-propelled particles endowed with nematic alignment rules are expected to display the same large-scale phenomenology. The existence of a centrifugal force does not rely on the direction of the individual trajectories. Shaken rods, concentrated suspensions of bacteria or motile biofilaments, among other possible realizations, are expected to have a similar phase behaviour. Quantitative local analysis of their spatial patterns^10^,^12^,^15^,^16^,^17^ would make it possible to further test and elaborate our understanding of the structure of confined active matter. For instance, the polar order found in confined bacteria is destroyed upon increasing the size of the confinement. The analysis of the spacial distribution of the bacteria could be used to gain insight on the symmetries and the magnitude of the additional interactions mediated by the host fluid, which are responsible for the emergence of bacterial turbulence^17^.

In conclusion, we take advantage of a model experimental system where ensembles of self-propelled colloids with well-established interactions self-organize into macrosopic vortices when confined by circular geometric boundaries. We identify the physical mechanism that chiefly dictates this emergent behaviour. Thanks to a combination of numerical simulations and analytical theory, we demonstrate that orientational couplings alone cannot account for collective circular motion. Repulsion between the motile individuals is necessary to balance the centrifugal flow intrinsic to any ordered active fluid and to stabilize heterogeneous yet monophasic states in a broad class of active fluids. A natural challenge is to extend this description to the compact vortices observed in the wild, for example, in shoals of fish. In the absence of confining boundaries, the centrifugal force has to be balanced by additional density-regulation mechanisms^35^,^36^. A structural investigation akin to the one introduced here for roller vortices could be a powerful tool to shed light on density regulation in natural flocks, which remains to be elucidated.

## Methods

### Experiments

We use fluorescent PMMA colloids (Thermo scientific G0500, 2.4 μm radius), dispersed in a 0.15 mol l^−1^ AOT/hexadecane solution. The suspension is injected in a wide microfluidic chamber made of double-sided scotch tapes. The tape is sandwiched between two ITO-coated glass slides (Solems, ITOSOL30, 80 nm thick). An additional layer of scotch tape including a hole having the desired confinement geometry is added to the upper ITO-coated slide. The holes are made with a precision plotting cutter (Graphtec robo CE 6,000). The gap between the two ITO electrodes is constant over the entire chamber *H*=220 μm. The electric field is applied by means of a voltage amplifier (Stanford Research Systems, PS350/5000 V-25 W). All the measurements were performed 5 min after the beginning of the rolling motion, when a steady state was reached for all the observables.

The colloids are observed with a × 4 microscope objective for particle tracking, particle imaging velocimetry (PIV) and number-density measurements. High-speed movies are recorded with a CMOS camera (Basler ACE) at a frame rate of 190 fps. All images are 2,000 × 2,000 8-bit pictures. The particles are detected to sub-pixel accuracy and the particle trajectories are reconstructed using a MATLAB version of a conventional tracking code^37^. The PIV analysis was performed with the mpiv MATLAB code. A block size of 44 μm was used.

### Numerical simulations

The simulations are performed by numerically integrating the equations of motion (equations 1 and 2). Particle positions and rolling directions are initialized randomly inside a circular domain. Integration is done using an Euler scheme with an adaptive time step *δt*, and the diffusive term in the equation for the rotational dynamics is modelled as a Gaussian variable with zero mean and with variance 2*D*/*δt*. Steric exclusion between particles is captured by correcting particle positions after each time step so as to prevent overlaps. Bouncing off of particles at the confining boundary is captured using a phenomenological torque that reorients the particles towards the centre of the disc; the form of the torque was chosen so at the reproduce the bouncing trajectories observed in the experiments.

## Additional information

**How to cite this article:** Bricard, A. *et al.* Emergent vortices in populations of colloidal rollers. *Nat. Commun.* 6:7470 doi: 10.1038/ncomms8470 (2015).

## Supplementary Material

Supplementary Information Supplementary Figures 1-3, Supplementary Notes 1-2 and Supplementary References

Supplementary Movie 1 Epifluorescence movie of a dilute ensemble of colloidal rollers exploring a circular chamber. Rc=1 mm. Packing Fraction: 0.3%. Movie recorded at 100 fps, played at 25 fps.

Supplementary Movie 2 Emergence of a macroscopic vortex pattern. Packing fraction: 3.6%. Rc=1 mm. Epifluorescence movie recorded at 100 fps, played at 11 fps. At t=3 s, the electric field is turned on and the rollers start propelling.

Supplementary Movie 3 Numerical simulation of a population of rollers showing the formation of an axisymmetric vortex. Packing fraction: 10%, range of repulsive forces: b=5a.

Supplementary Movie 4 Numerical simulation of a population of rollers showing the formation of a finitesized swarm. Packing fraction: 4.5%, range of repulsive forces: b=2a.
