# Supplementary material for: Emergent vortices in populations of colloidal rollers
Source: Nat Commun. 2015 Jun 19;6:7470. doi: 10.1038/ncomms8470 (PMC4557359; doi:10.1038/ncomms8470)
Supplement: Supplementary Information — Supplementary Figures 1-3, Supplementary Notes 1-2 and Supplementary References [file ncomms8470-s1.pdf]

Supplementary Information

Supplementary Figures

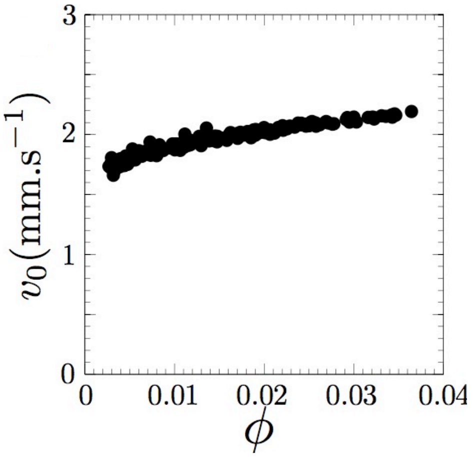

Supplementary Figure 1 | Variation of the roller velocity with the local area fraction. $E/E_Q = 1.4$. As ϕ varies from 10^{-2} to 4×10^{-2}, v_0 only increases by $\sim 10\%$.

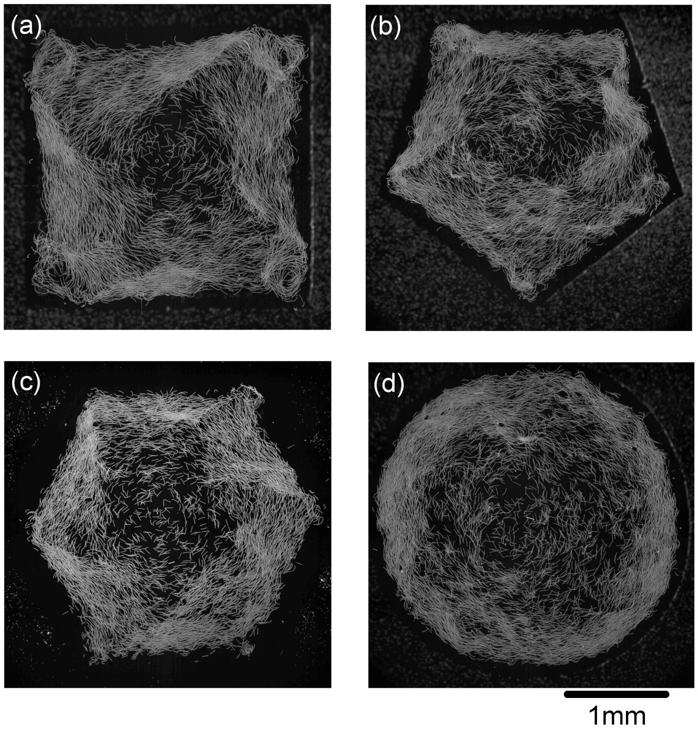

Supplementary Figure 2 | Superimposed pictures of colloidal rollers forming a vortex patterns in polygonal geometries. Packing fraction: $\phi_0 = 3 \times 10^{-2}$. $E/E_Q = 1.4$. The self organization of the population is robust to the shape of the confinement. These pictures show that the same heterogeneous vortex pattern emerge in polygonal confinements which do not have a perfect rotational symmetry. The center of the vortex is dilute and less ordered that the outer region where polar order is pronounced. The shape of the polygonal geometries is reflected by the velocity and density profiles yet the salient features of the vortex is preserved.

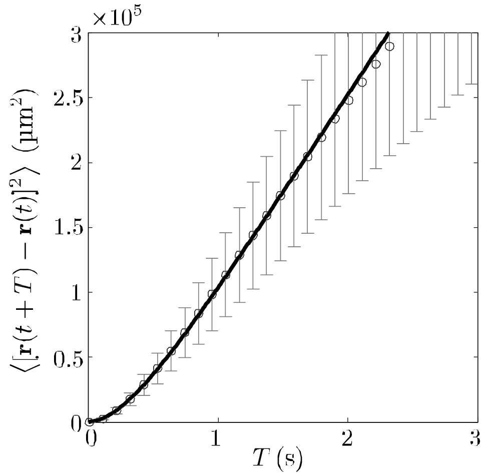

Supplementary Figure 3 | Mean-squared displacement of the rollers, $\langle[\mathbf{r}_i(t+T)-\mathbf{r}_i(t)]^2\rangle_{i,t}$ plotted as a function of the lag time T. Open symbol: experiments (error bars 1SD). Full line: theoretical prediction.

Supplementary Notes

Supplementary Note 1 | Dynamics of a single roller

At low packing fraction, $\phi_0 \ll \phi^*$, the colloids behave as non-interacting persistent random walkers. Their motion is described by:

$$\partial_t \mathbf{r}_i = v_0 \hat{\mathbf{p}}_i = v_0(\cos\theta_i, \sin\theta_i), \tag{1}$$

$$\partial_t \theta_i = \xi_i(t), \tag{2}$$

where ξ_i is a white noise with zero mean and variance $\langle\xi_i(t)\xi_i(t')\rangle = 2D\delta(t-t')$. The velocity autocorrelation function decays exponentially as

$$\langle v_0\hat{\mathbf{p}}_i(t+T)\cdot v_0\hat{\mathbf{p}}_i(t)\rangle = v_0^2 \mathrm{e}^{-DT}, \tag{3}$$

and the mean-squared displacement is given by:

$$\langle[\mathbf{r}_i(t+T)-\mathbf{r}_i(t)]^2\rangle = 2\frac{v_0^2}{D^2}\left(DT - 1 + \mathrm{e}^{-DT}\right). \tag{4}$$

The later expressions are used to fit the experimental data, Fig. 2E (main text) and Supplementary Figure 3 below, and provide the following values for the particle speed and diffusivity:

$$v_0 = 493 \pm 17\,\mu\mathrm{m.s}^{-1}, \tag{5}$$

$$D^{-1} = 0.31 \pm 0.02\,\mathrm{s}. \tag{6}$$

Supplementary Note 2 | Population of interacting rollers

Microscopic model. In [1], we have theoretically described the microscopic dynamics of a population of colloids rolling on a conducting surface. We briefly summarize this model. A colloid powered by the Quincke mechanism, when rotating close to a wall, exchanges momentum with this solid surface and translates at a speed given by

$$v_0 = \frac{a\tilde{\mu}_t}{\mu_r \tau}\sqrt{\left(\frac{E_0}{E_Q}\right)^2 - 1}. \tag{7}$$

E_Q is the critical electric field below which the particle does not rotate. The Maxwell-Wagner time τ characterizes the dynamics of the electric charges at the colloid surface, which are responsible for the Quincke instability. μ_r and $\tilde{\mu}_t$ are mobility coefficients accounting for the viscous drag exerted by the liquid in the vicinity of solid wall that depend logarithmically on the distance to the solid wall [1]. In order to account for the roller-roller interactions at long distances, we have computed the electrostatic and hydrodynamic fields induced by the motion of a colloid. Assuming pairwise additive interactions, we have shown that these fields promote the alignement of the velocity of neighboring particles. Within this framework, the speed of the colloids is taken to be a constant as it relaxes to v_0 much faster than the typical timescale of the orientation dynamics. In addition to these far-field couplings, we model the short-distance repulsion between colloids by an effective hard-core potential. The resulting equations of motion are given by Eqs. [1]– [3] in the main text, where the functions $A(r)$, $B(r)$, $C(r)$ are given by:

$$A(r) = A_1\left(\frac{a}{r}\right)^3\Theta(r) + A_2\left(\frac{a}{r}\right)^5\Theta(r), \tag{8}$$

$$B(r) = B_1\left(\frac{a}{r}\right)^4\Theta(r), \tag{9}$$

$$C(r) = C_1\left[\frac{a}{2H}\left(\frac{a}{r}\right)^2 + \left(\frac{a}{r}\right)^3\right]\Theta(r) + C_2\left(\frac{a}{r}\right)^5\Theta(r). \tag{10}$$

These electrostatic and hydrodynamic couplings are exponentially screened over a distance set by the channel height H (see Fig. 1A, main text). For sake of simplicity, we approximate the screening function $\Theta(r)$ by the step function $\Theta(r) = 1$ if $r \leq H/\pi$ and $\Theta(r) = 0$ otherwise. In addition, the coefficients of the above functional forms are given by:

$$A_1 = 3\tau^{-1}\tilde{\mu}_s, \tag{11}$$

$$A_2 = 9\tau^{-1}\left(\frac{\mu_\perp}{\mu_r} - 1\right)\left[\chi^\infty + \frac{1}{2}\left(1 - \frac{E_Q^2}{E_0^2}\right)\right], \tag{12}$$

$$B_1 = 6\tau^{-1}\left(\frac{\mu_\perp}{\mu_r} - 1\right)\sqrt{\frac{E_0^2}{E_Q^2} - 1}\left[\left(\chi^\infty + \frac{1}{2}\right)\frac{E_Q^2}{E_0^2} - \chi^\infty\right], \tag{13}$$

$$C_1 = A_1, \tag{14}$$

$$C_2 = \frac{5}{3}A_2. \tag{15}$$

μ_r and $\mu_\perp$ are mobility coefficients which only depend on the viscosity of the liquid and the gap d between the colloid and the surface. χ^∞ depends on the dielectric permittivities ϵ_l and ϵ_p of the liquid and of the particles: $\chi^\infty = (\epsilon_p - \epsilon_l)/(\epsilon_p + 2\epsilon_l)$ The derivation of this model is provided in the supplementary informations of [1].

Estimation of the simulation parameters. In order to perform numerical simulations relevant to our experimental conditions, we have estimated the coefficients of the equations of motion as follows. The speed v_0 and the rotational diffusivity D have been deduced from the single-particle dynamics, Eqs. (5) and (6). The threshold electric field E_Q is measured experimentally as the critical value at which the colloids start moving. We use typical values for the dielectric permittivities of hexadecane ($\epsilon_l = 2.2\epsilon_0$) and PMMA colloids ($\epsilon_p = 2.6\epsilon_0$) [2] to evaluate $\chi^\infty = 0.06$. The mobility coefficients are estimated. We assume the distance between a particle and the surface to be $d \sim 50$ nm. Although this parameter is not controlled precisely, it only yields small corrections to the mobility coefficients in the limit $d \ll a$, and weakly impacts the particle dynamics. Using the expressions derived in [3, 4, 5, 6], we find $\tilde{\mu}_s = 0.30$, $\mu_\perp/\mu_r = 1.6$ and $\tilde{\mu}_t/\mu_r = 8.7\times10^{-2}$
Finally, the Maxwell-Wagner time τ was calculated from Eq. (7): $\tau = 0.29$ ms. As a result, we obtain the following values for the microscopic coefficients: $A_1 = B_1 = C_1 = 0.9\tau^{-1}$, $A_2 = 1.0\tau^{-1}$, and $C_2 = 1.7\tau^{-1}$.

Supplementary References

[1] Bricard A., Caussin J.-B., Desreumaux N., Dauchot O. & Bartolo D. Emergence of macroscopic directed motion in populations of motile colloids. *Nature* 503, 95– 98 (2013).

[2] Pannacci N., Lemaire E. & Lobry L. DC conductivity of a suspension of insulating particles with internal rotation. *Eur. Phys. J. E* **28**, 411– 417 (2009).

[3] Goldman A. J., Cox R. G. & Brenner H. Slow viscous motion of a sphere parallel to a plane wall – I Motion through a quiescent fluid. *Chem. Eng. Sci.* **22**, 637– 651 (1967).

[4] Goldman A. J. , Cox R. G. & Brenner H. Slow viscous motion of a sphere parallel to a plane wall – II Couette flow. *Chem. Eng. Sci.* **22**, 653– 660 (1967).

[5] O' Neill M. E. & Stewartson K. On the slow motion of a sphere parallel to a nearby plane wall *J. Fluid Mech.* **27**, 705– 724 (2006).

[6] Liu Q. & Prosperetti A. Wall effects on a rotating sphere *J. Fluid Mech.* **657**, 1– 21 (2010).

[7] Desreumaux N., Caussin J.-B., Jeanneret R., Lauga E. & Bartolo D. Hydrodynamic fluctuations in confined emulsions. *Phys. Rev. Lett.* **111**, 118301 (2013).
